# Stomatal development and orientation: a phylogenetic and ecophysiological perspective

**DOI:** 10.1093/aob/mcad071

**Published:** 2023-06-08

**Authors:** Paula J Rudall

**Affiliations:** Royal Botanic Gardens, Kew, Richmond, UK

**Keywords:** Auxin signalling, cellular differentiation, leaf fossils, parasitic plants, transverse stomata, xeromorphy

## Abstract

**Background:**

Oriented patterning of epidermal cells is achieved primarily by transverse protodermal cell divisions perpendicular to the organ axis, followed by axial cell elongation. In linear leaves with parallel venation, most stomata are regularly aligned with the veins. This longitudinal patterning operates under a strong developmental constraint and has demonstrable physiological benefits, especially in grasses. However, transversely oriented stomata characterize a few groups, among both living angiosperms and extinct Mesozoic seed plants.

**Scope:**

This review examines comparative and developmental data on stomatal patterning in a broad phylogenetic context, focusing on the evolutionary and ecophysiological significance of guard-cell orientation. It draws from a diverse range of literature to explore the pivotal roles of the plant growth hormone auxin in establishing polarity and chemical gradients that enable cellular differentiation.

**Conclusions:**

Transverse stomata evolved iteratively in a few seed-plant groups during the Mesozoic era, especially among parasitic or xerophytic taxa, such as the hemiparasitic mistletoe genus *Viscum* and the xerophytic shrub *Casuarina*, indicating a possible link with ecological factors such as the Cretaceous CO_2_ decline and changing water availability. The discovery of this feature in some extinct seed-plant taxa known only from fossils could represent a useful phylogenetic marker.

## INTRODUCTION

Polarized development in land plants operates at multiple structural levels, from individual cells to tissues and entire organs. In stems, pavement epidermal cells typically exhibit axial alignment, parallel to the organ axis and the major veins; similarly, they are arranged longitudinally in leaves with parallel venation, such as those of grasses and conifers. This oriented patterning of epidermal cells is achieved primarily by transverse protodermal cell divisions perpendicular to the organ axis, followed by axial cell elongation (Apostolakos *et al.*, 2018). Data from a diverse range of sources have long indicated a pivotal role for the plant growth hormone auxin (indole-3-acetic acid) in establishing polarity and chemical gradients that enable such differentiation. Leaf shapes and venation patterns are correlated with directional auxin transport during critical growth periods before cell expansion (Benkova *et al.*, 2003; Scarpella *et al.*, 2010; Sawchuk *et al.*, 2013; McKown and Bergmann, 2020; Perico *et al.*, 2022). Auxin is transported via the phloem away from its source in young apical regions and margins through the plant body towards the root apex and, more locally, by cell-to-cell directional transport (Benkova *et al.*, 2003; Petrášek and Friml, 2009).

At the precise cellular level, directional auxin distribution is enabled by transport proteins, most notably those generated by the PIN-FORMED (PIN) and AUX1/LAX gene families of auxin efflux carriers (Adamowski and Friml, 2015; Kasprzewska *et al.*, 2015). As leaves grow, acropetal flux along the developing vascular bundles results in auxin accumulation at the vein tips, which are mostly located at the leaf apex in species with parallel venation, such as grasses, and at the margins or leaf areoles in other taxa. Multiple PIN mutant combinations can display abnormal stomatal formation, often resulting in contiguous stomatal guard cells (GCs); similar perturbations can result from application of toxins that disrupt auxin transport (Balcerowicz and Hoecker, 2014). Abnormal stomata can also occur at sites with high auxin levels, such as hydathodes and anthers. Hydathodes at leaf margins are often associated with modified and enlarged stomata (Torii, 2021). Abnormal stomata can occur on the stamen connectives in taxa such as *Momordica* (Cucurbitaceae), often exhibiting proliferated GCs (Rao and Ramayya, 1967).

Stomatal development is highly specialized within the epidermal cell matrix (for terms, see Table 1). At maturity, each stomatal pore is flanked by a pair of GCs derived from symmetric division of a guard-mother cell (GMC). In turn, GMCs are typically established by meristemoids, which are localized cells that undergo asymmetric (polarized) mitoses resulting in specialized differentiation (Bünning, 1952; Sachs, 1991). Each asymmetric mitosis results in a smaller cell and a larger stomatal-lineage ground cell (SLGC); the smaller cell either forms a GMC or undergoes a further asymmetric mitosis that is termed an amplifying division (Payne, 1979; Rudall *et al.*, 2013). Asymmetric mitoses are predetermined by the cytoskeleton of the primary nucleus. Cellular polarization, microtubules and localization of gamma-tubulin all play important roles throughout cell morphogenesis (Muroyama *et al.*, 2020; Spiegelhalder and Raissig, 2021). Stomatal development is controlled by a series of basic helix–loop–helix (bHLH) transcription factors: *SPEECHLESS* (*SPCH*), *MUTE* and *FAMA* (Ohashi-Ito and Bergmann, 2006; Bergmann and Sack, 2007; MacAlister *et al.*, 2007; Pillitteri *et al.*, 2007). Asymmetric mitoses in the stomatal cell lineage are initiated and promoted by *SPCH*; the transition from a meristemoid to a GMC is triggered by its paralogue *MUTE*, and *FAMA* has an important role in establishing GC formation.

**Table 1.** Glossary

Term	Definition
Amplifying division	Further asymmetric division of a meristemoid that creates two daughter cells: an SLGC and a meristemoid or GMC
Guard cells (GCs)	Pair of cells that together delimit the stomatal pore
Guard-mother cell (GMC)	Final stomatal precursor cell; divides symmetrically to form a pair of guard cells (GCs)
Lateral subsidiary cell (LSC)	Modified neighbour cell located laterally adjacent to a guard cell; can be mesogenous or perigenous
Meristemoid	Specialized precursor cell that typically divides asymmetrically
Mesogenous neighbour cell (or subsidiary cell), also termed stomatal-lineage ground cell (SLGC)	Neighbour cell derived from the same lineage as the GCs
Perigenous neighbour cell (or subsidiary cell)	Neighbour cell derived from a different cell lineage from the GCs (e.g. grass LSCs)
Quartet pre-patterning	Protodermal cells in groups of four, roughly arranged in a square or rectangle, resulting from development whereby each cell divides symmetrically across its narrowest width, usually perpendicular to the previous division

**Fig. 1.**
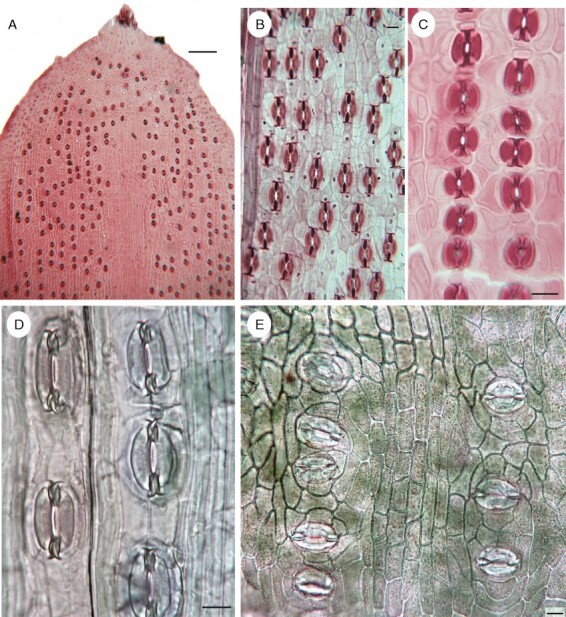
Photomicrographs of abaxial surfaces of gymnosperm leaves (all conifers, except B, Gnetales), taken from prepared microscope slides (in A–C) or from differential interference contrast images of cleared paradermal sections of living material (in D, E). Stomata are longitudinal (in A–D) or transverse (in E). (A) *Taxus baccata*. (B) *Ephedra gerardiana*. (C) *Podocarpus nivalis*. (D) *Araucaria bidwillii*. (E) *Agathis dammara*. Scale bars: 50 µm in A; 20 µm in B–E.

**Fig. 2.**
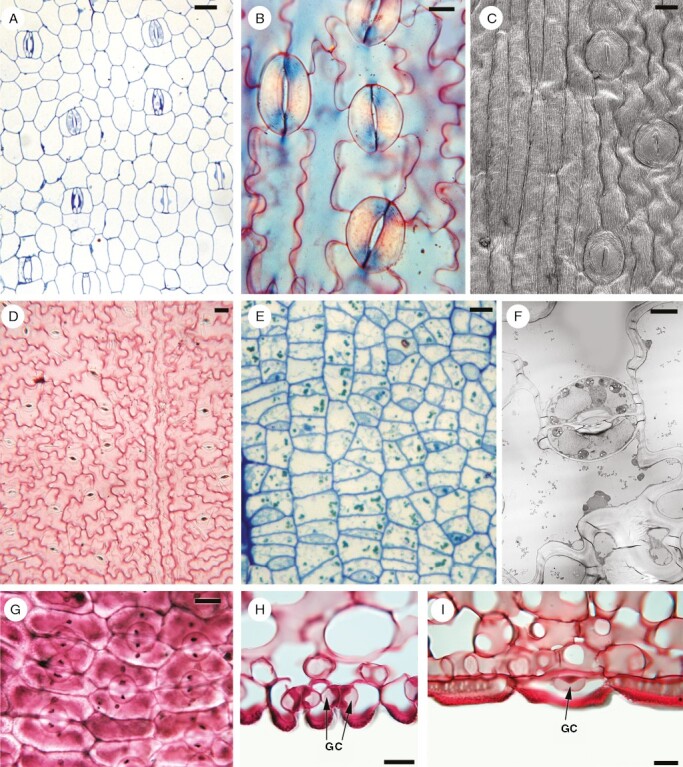
Photomicrographs of monocot leaves. (A–G) Surface views of leaf epidermis with veins oriented in a similar manner: (A–C) longitudinal stomata (GCs oriented parallel to primary vasculature); and (D–I) transverse stomata. (A) *Eichhornia crassipes* (Pontederiaceae), Light micrograph (LM) of surface with mature stomata, each with a pair of LSCs. (B) *Cardiocrinum giganteum* (Liliaceae), LM abaxial surface with stomata lacking subsidiary cells (SCs). (C) *Lilium kesselringhianum* (Liliaceae), scanning electron microscope image of abaxial surface with mature stomata lacking SCs. (D–F) *Lapageria rosea* (Philesiaceae); (D) LM mature abaxial epidermis with transverse stomata lacking SCs; (E) LM abaxial epidermis at GMC stage; and (F) transmission electron microscope image of a single stomatal pore. (G–I) *Philesia magellanica* (Philesiaceae): (G) surface with transverse stomata; (H) longitudinal section showing two stomata (GCs indicated); and (I) transverse section showing one stoma below adjacent epidermal cell, with a single GC visible (indicated). Scale bars: 20 µm.

**Fig. 3.**
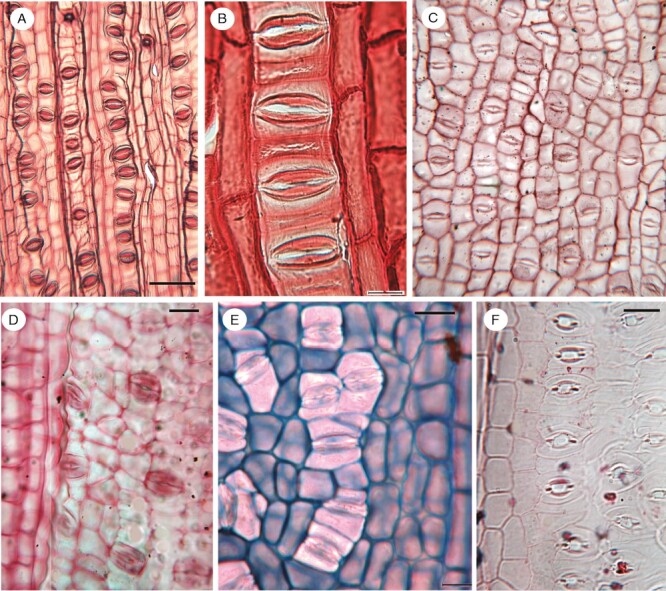
Species with stomata oriented either transversely or obliquely with respect to the longitudinal axis. (A, B) *Cassytha glabella* (Lauraceae). (C) *Viscum articulatum* (Santalaceae or Viscaceae). (D) *Thesium canariense* (Santalaceae). (E) *Santalum album* (Santalaceae). (F) *Casuarina equisetifolia* (Casuarinaceae). Scale bars: 20 µm.

Typically, in linear leaves with parallel venation, such as those of some conifers and many monocots (Figs 1A–D and 2A–C), the long axes of the stomatal GCs (and hence the long axes of the stomatal pores) are regularly aligned with the veins. However, adjustments (sometimes relatively minor) in the relative timing and polarity of GMC and GC establishment can result in modifications in stomatal patterning, including transversely oriented stomata (Figs 1E, 2D–I and 3). In a few cases (discussed below), such mutations can be non-lethal and have become fixed during evolution.

The number and orientation of the asymmetric mitoses that precede GMC formation are often taxon specific and can regulate the angles of the stomatal pores. Exceptions to the general rule of an axially oriented stomatal pore have been reviewed in earlier literature (Smith, 1935; Butterfass, 1987), although these studies precede modern molecular classifications. More detailed comparison in an updated phylogenetic context is important for understanding the potential homologies and ecophysiological significance of transverse stomatal orientation, which is not only highly iterative, but can also differ in developmental details. Improvements in our understanding of the physiological and morphogenetic factors that underlie stomatal development prompt re-examination of their significance, especially in light of recent discoveries of fossil taxa with transverse stomatal orientation. Technological developments in areas of plant research ranging from phylogenetics to finely tuned gene-expression studies enhance our understanding of the associations between genes, biochemistry and functional traits.

This review examines comparative and developmental data on stomatal patterning in a broad phylogenetic context. Focusing on the co-ordinated morphogenetic factors that influence polarized differentiation, it examines the evolutionary and ecophysiological significance of particular aspects of stomatal patterning, especially GC orientation.

## LONGITUDINAL GUARD-CELL ORIENTATION: AUXINS AND MICROTUBULES

The very high frequency of longitudinal stomatal alignment in stems and linear leaves indicates that the regulatory factors that promote this feature are strongly constrained. To achieve longitudinal orientation of the GCs and thus the stomatal pore, a remarkable localized developmental polarity shift occurs within the dividing GMC. In contrast with the other protodermal cells, which mostly divide transversely, the GMC divides longitudinally, so that the pore is parallel to the organ axis (Galatis and Apostolakos, 2004; Apostolakos *et al.*, 2018; Spiegelhalder and Raissig, 2021). It does this by repositioning of the microtubule arrays during GMC mitosis.

The cellular reorientation that occurs during GMC mitosis has been described in detail in both monoplastidic non-seed plants (in the lycophyte *Selaginella*) and in seed plants, which are polyplastidic (Pickett-Heaps *et al.*, 1999). Plastid reorientation and division precede wall formation. In *Selaginella*, the single GMC plastid divides into two daughter plastids that migrate to opposite poles and rotate in the cell by extensive reshuffling of cytoskeletal arrays (Brown and Lemmon, 1985). Among seed plants, repositioning of microtubule arrays during GMC mitosis has been documented in several monocots. Monocot leaves, which are essentially linear in outline with parallel venation, represent a useful model for this feature; in most monocots (including all commelinid monocots), the epidermal cells and stomatal pores are oriented parallel to the major veins (Stewart and Dermen, 1979; Rudall *et al.*, 2017; Fig. 2A–C). In mitotic GMCs of *Allium*, *Iris* and *Tradescantia*, the developing cell plate is oriented obliquely at first, but from metaphase onwards it rotates to a longitudinal position (Palevitz and Hepler, 1974; Butterfass, 1987; Tarkowska *et al.*, 1987). In grasses, such as *Avena sativa*, nuclear migration occurs in the long cells adjacent to the GMC, followed by cell division and lateral subsidiary cell (LSC) formation. Subsequently, within the GMC there is a 90° shift in microtubule orientation and initiation of a longitudinal preprophase band (Kaufman *et al.*, 1970; Mullinax and Palevitz, 1989).

Developmental studies in *Arabidopsis* have shown that the transition from a meristemoid (which divides asymmetrically) to a GMC (which divides symmetrically) is accompanied by a localized intracellular depletion in auxin levels in the smaller daughter cell of an asymmetric mitosis, resulting in transformation of this cell into a GMC (Le *et al.*, 2014). The timing of this effect is crucial, not only in eudicots such as *Arabidopsis*, but also in other angiosperms. In monocot taxa that characteristically possess LSCs (e.g. commelinid monocots such as grasses and *Tradescantia*), auxin accumulates in the contact regions between the premitotic GMC and its lateral contact cells in adjacent cell lineages, inducing asymmetric mitoses that result in LSC formation (Galatis and Apostolakos, 2004; Balcerowicz and Hoecker, 2014; Livanos *et al.*, 2015; Apostolakos *et al*., 2018). Thus, these LSCs are perigenous cells, formed from a different cell lineage to the GCs (Table 1). In commelinid monocots, the bHLH transcription factor *MUTE* has apparently adopted a novel secondary role in generating lateral polarization and asymmetric division of cells in adjacent cell files, resulting in a four-celled paracytic stomatal complex (Spiegelhalder and Raissig, 2021). Achieving this fine balance relies on localized expression of PIN auxin transporters in specific regions of particular cell types to enable dynamic auxin gradients (Zhang *et al.*, 2020).

The finely-tuned balance in cell morphogenesis is rarely disrupted, although an early experiment in treatment of grass cotyledons with the chemical mercaptoethanol (Stebbins *et al.*, 1967) resulted in major effects, such as failure of LSC formation and failure of GMC spindle reorientation. A more recent study found that grass *Bdmute* mutants lacked LSCs and displayed some misoriented GMC divisions and aborted GCs, indicating a significant role for *MUTE* in patterning of the stomatal complex in grasses (Raissig *et al.*, 2017).

Likewise, in leaves and stems of many other tracheophytes, the GMCs typically divide so that GC orientation is either parallel to the midvein or parallel to the major secondary veins. For example, in most fern leaves, the long axis of the stomatal pore is oriented parallel to the secondary veins, which are attached obliquely to the central primary vein (Van Cotthem, 1970). Stomatal pores are also aligned longitudinally in photosynthetic stems of the pteridophyte genera *Psilotum* and *Equisetum* (Cullen and Rudall, 2016) and in the parallel-veined leaves of many conifers (Florin, 1931, 1933; Fig. 1A–C). In all these taxa (as in monocots), each meristemoid gives rise to a GMC and an SLGC in the same axial cell file, although *Equisetum* is exceptional in that a meristemoid divides twice to produce three longitudinally aligned cells: a GMC flanked by two mesogenous subsidiary cells (Cullen and Rudall, 2016).

## OTHER MODES OF GUARD-CELL ORIENTATION

Not all taxa display predominantly longitudinal or transverse stomatal orientation. In some taxa, stomata orientation is apparently not controlled by a radical reshifting of microtubule arrays in dividing GMCs, but is determined at an earlier stage in the stomatal ontogenetic pathway. For example, in leaves of many reticulate-veined eudicots such as *Arabidopsis*, stomata are oriented randomly with respect to the major veins and occur in non-contiguous groups or clusters in the interveinal areolar regions. These stomata are each formed following a series of one to three asymmetric mitoses; the resulting cell lineage is organized in an inward spiral arrangement due to the excentric orientation of each amplifying division (Zhao and Sack, 1999; Lau and Bergmann, 2012; Muroyama *et al.*, 2020). Thus, mature stomatal pore orientation depends on various factors, including meristemoid shape and the number of amplifying divisions (Lucas *et al.*, 2006). In stems of *Arabidopsis*, the stomata follow a similar developmental pathway, but are ultimately aligned longitudinally as a result of axial orientation of the meristemoids and their amplifying divisions, followed by GMC division and longitudinal elongation of pavement cells (Bhave *et al.*, 2009).

Such repetitive amplifying divisions are rare in other taxa with reticulate venation, such as many magnoliids and ANA-grade species and the relictual living gymnosperm *Gnetum gnemon*. In these taxa, stomata can originate from ‘quartet’ pre-patterning of protodermal cells, resulting in irregular orientations that become increasingly chaotic as the leaf enlarges (Carpenter, 2005; Rudall and Knowles, 2013; Rudall and Rice, 2019). Differences in stomatal orientation result primarily from differences in protodermal cell shapes and division orientation at critical stages in leaf expansion. In magnoliids, stomatal complexes develop mostly from rectangular meristemoids that form linear triads consisting of a GMC flanked by two equal SLGCs, although other categories are also present, including triangular meristemoids that produce three unequal SLGCs on the three sides of the triangle (Rudall, 2023).

## TRANSVERSE GUARD-CELL ORIENTATION

**Fig. 4.**
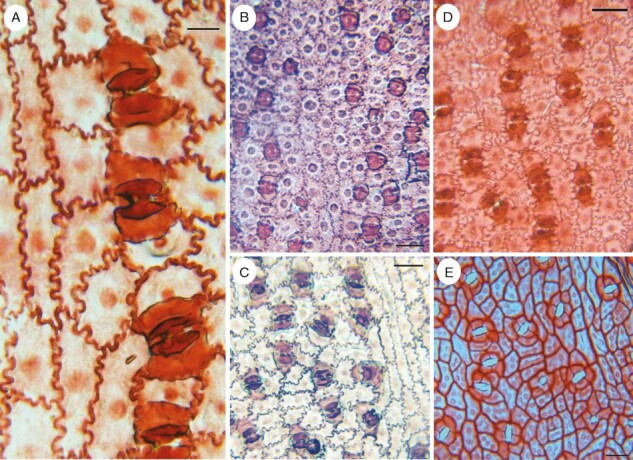
Photomicrographs of abaxial leaf surfaces of fossil taxa. (A–C) *Dictyozamites johnstrupii* (Bennettitales), transverse stomata. (D) *Otozamites bornholmiensis* (Bennettitales), transverse stomata. (E) *Androvettia* sp. (conifer), stomata variously oriented. Scale bars: 20 µm in A; 50 µm in B–E.

Before discussing the factors that control non-random GC orientation, it is useful to compare examples of taxa with transverse stomata and place them in a phylogenetic context. Species that possess predominantly transverse stomatal alignment are relatively rare and dispersed widely across phylogenies, not only within angiosperms, but also within conifers and other tracheophyte groups. This feature was reviewed by Smith (1935) and Butterfass (1987), in studies that pre-dated modern classifications based on molecular phylogenetics. These authors observed a possible correlation between transverse stomata and various life forms, notably xeromorphy (including halophytes, desert plants and succulents) and parasitism, including hemiparasites. Leaves (or pinnae) of taxa with transverse stomata can be assigned tentatively to two main types: (1) reduced, scale-like leaves that are often triangular and imbricate, such as those of some parasitic or xerophytic angiosperms; and (2) linear, strap-like leaves, such as those of some monocot families with a climbing habit (e.g. Philesiaceae; Fig. 2D–I) and the extinct order Bennettitales (Fig. 4A–D). In species with transverse stomata, the guard cells appear longitudinal in transverse sections and transverse in longitudinal sections (Fig. 2H, I).

The floating aquatic water fern *Azolla* represents an exceptional example of transverse stomata. During leaf development in this genus, a transverse preprophase band forms within the GMC, but the ensuing cell plate breaks down near the cell margins, resulting in a single ring-like binucleate GC (Busby and Gunning, 1984). In general, stomata are absent from aquatics with submerged leaves (e.g. the submerged aquatic family Podostemaceae) and borne on the upper (adaxial) surface of floating leaves, although the stomata are vestigial in the floating water fern *Salvinia* in which the GCs are reduced to mere extensions of wall material, and the aperture remains fixed open (Croxdale, 1978). In the floating leaves of *Nymphaea* and *Cabomba* (waterlilies of the ANA-grade family Nymphaeaceae), stomatal development differs from that of most other tracheophytes in that the protodermal cells give rise directly to GMCs, with no asymmetric divisions (Rudall and Knowles, 2013). In these taxa, stomata are mostly oriented in a similar manner on the leaf, probably following the direction of leaf expansion.

### Transverse stomata in angiosperms

**Fig. 5.**
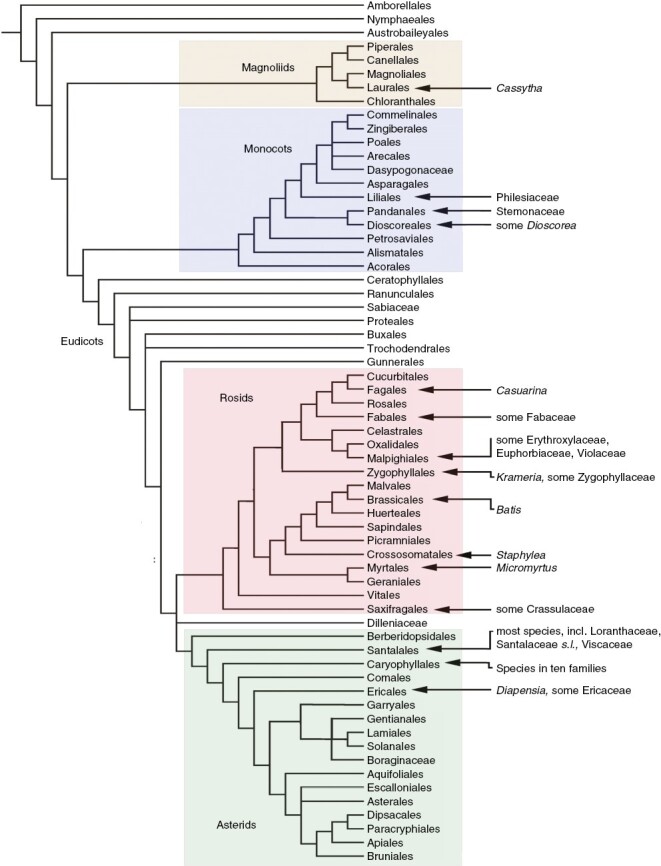
Tree diagram showing relationships among angiosperm orders, based on the classification of the Angiosperm Phylogeny Group III (2009), showing distribution of families that include species with transverse stomata (see main text for details).

Among extant angiosperms (Fig. 5), transverse stomata are rare in the ANA-grade and magnoliid clade (Rudall and Knowles, 2013; Rudall, 2023). In the magnoliid clade, the sole reported exception is a hemiparasitic climber, *Cassytha* (Lauraceae), in which the leaves are highly reduced and scale-like, and the stomata are borne on long stems that extend rapidly and twine around the host plant, producing invasive haustoria at the closest contact points (Heide-Jørgensen, 1991). In this genus, the stomatal pores are always oriented transversely with respect to the axis, and the LSCs are mesogenous cells that are initiated in the same cell file as the GCs (Fig. 3A, B), in contrast to grass LSCs. The stomata are organized in rows of several closely adjacent stomatal complexes, each separated by a single narrow intervening cell. In other Lauraceae, stomata display diverse orientations (Rudall, 2023).

Among eudicots, transverse stomata are not recorded in early-divergent lineages, but several examples are known in the rosid and asterid clades (Fig. 3). Most asterids with transverse stomata belong to the orders Caryophyllales and Santalales, with a few additional records in Ericales (Fig. 5). Species of Caryophyllales with transverse stomata include leaf and stem succulents and other xerophytes or unusual life forms [e.g. many Amaranthaceae, *Carpobrotus*, many epiphytic Cactaceae (tribe Hylocereeae), Didiereaceae, *Frankenia*, some *Nepenthes*, some Polygonaceae, Portulacaceae and *Tamarix*]. In Santalales, transverse stomata mostly occur in hemiparasites, such as *Comandra*, some Loranthaceae, Opiliaceae, *Thesium*, *Viscum* and many Santalaceae *sensu stricto*, although not in holoparasites, such as *Cuscuta*. Wilson and Calvin (2003) reported transverse stomata in all genera of Viscaceae and Eremolepidaceae and some Santalaceae; they suggested that anatomical characters such as a cuticular epithelium and transverse stomata could be taxonomically informative in these families. In the hemiparasitic shrub *Viscum articulatum* (Fig. 3C), the stomata are paracytic with mesogenous LSCs in the same cell files; they are densely arranged, with only a few short intervening cells between stomatal complexes.

Among scattered examples in the rosid clade, perhaps the most noteworthy is the Australian genus *Casuarina*, in which the leaves are minute and scale-like and the stomata are borne in longitudinal furrows on both the leaves and the green photosynthetic stems. As in *Cassytha* and *Thesium*, the paracytic stomatal complexes in *Casuarina* are oriented transversely in axial cell files, with each complex separated by a single narrow intervening cell (Fig. 3F). A detailed ontogenetic study of *Casuarina* showed that during leaf epidermal development, each GMC is flanked by a pair of SLGCs, but there is no reorientation of the cell plate during GMC mitosis (Pant *et al.*, 1975).

In monocots, transverse stomata are rare, but they characterize species of a few families that possess relatively broad leaves and a climbing habit (Conover, 1991; Rudall *et al.*, 2017). They occur in two climbing families of the order Liliales [Philesiaceae (*Lapageria* and *Philesia*; Fig. 2D–I) and Smilacaceae (*Ripogonum* and *Smilax*)], a distribution that indicates a single origin of this feature in these closely related families. In contrast, other climbers in the order Liliales, such as Alstroemeriaceae, possess longitudinally oriented stomata. Stomata are also predominantly transverse in the climbing monocot family Stemonaceae (Pandanales) and either transverse or more randomly oriented in some climbing species of *Dioscorea* (Dioscoreales). Although true parasites are lacking in monocots, many species are either partly or entirely mycoheterotrophic; however, the stomata of monocot mycoheterotrophs are longitudinally oriented and often highly reduced in number.

Very few fossil angiosperms display transverse stomata. However, in Cretaceous fossil leaves of *Pseudoasterophyllites*, the stomata are widely separated and sparse but predominantly transverse, in contrast to its putative living relatives, Chloranthaceae and *Ceratophyllum* (Kvaček *et al.*, 2016). Stomata are absent from the submerged water plant *Ceratophyllum* and randomly oriented in Chloranthaceae (Carpenter, 2005).

### Transverse stomata in extant and fossil gymnosperms

Stomatal orientation that is predominantly transverse (as opposed to longitudinal or random) is rare in gymnosperms but characterizes a few groups (listed below). Although orientation is predominantly longitudinal in many gymnosperms, random stomatal orientation characterizes fossil and extant *Cycas* and the fossil cycad *Ctenis* (Griffith *et al.*, 2014; Erdei and Manchester, 2015). The sole parasitic extant conifer, *Parasitaxus usta* (Podocarpaceae), also has apparently random stomatal orientation within a chaotic matrix of epidermal cells of varying shapes and sizes, suggesting that development is irregular in this species (Stockey *et al.*, 1995). The anomalous Cretaceous fossil conifer *Androvettia* (Fig. 4E), which had highly reduced leaves on anomalous flattened frond-like stems, had stomata with apparently random orientation (Hueber and Watson, 1988; Raubeson and Gensel, 1991).

Among gymnosperms, predominantly transverse stomatal orientation occurs in the following taxa: (1) some Araucariaceae (both living and fossil); (2) the *Krassilovia* clade of Cretaceous seed plants; and (3) the extinct order Bennetittales.

#### Araucariaceae.

In most conifers (except Araucariaceae), leaf stomatal orientation is consistently longitudinal (Fig. 1A–C), although Florin’s (1931) detailed studies of epidermal structure development in conifers illustrated occasional (spasmodic) transverse stomata in *Taxodium* and *Cryptomeria* (both Cupressaceae *s.l.*).

Araucariaceae are an ancient conifer family consisting of only three extant genera (*Agathis*, *Araucaria* and *Wollemia*) plus several extinct genera known only from fossils. Leaves are persistent in Araucariaceae, and vegetative morphology differs between the three living genera. In *Agathis*, the leaves are opposite, petiolate and ovate or elliptical, compared with the spiral, sessile, sometimes triangular leaf morphology in *Araucaria* and variable leaf morphology in *Wollemia* (Cookson and Duigan, 1951; Chambers *et al.*, 1998). In all three genera, the stomata possess one or two rings of four to six subsidiary cells (often termed cyclocytic stomata or Florin rings). The stomatal pores are longitudinal in most species of *Araucaria* (although oblique or transverse in section *Eutacta*; Florin, 1931; Stockey and Ko, 1986) and predominantly transverse or oblique in *Agathis* (Kausik, 1976; Stockey and Taylor, 1978; Stockey and Atkinson, 1993; Pole, 2008; Fig. 1D, E). *Wollemia* is remarkable in that the stomata are transverse or oblique on leaves borne on the first (‘juvenile’) internodes of a branchlet (closest to the main axis) but longitudinal on later internodes (Cookson and Duigan, 1951; Chambers *et al.*, 1998). This ontogenetic contrast could reflect positional and temporal differences in auxin concentration, although this inference is speculative. A differential gradient in auxin levels in a single growth increment has been inferred previously in conifer leaves, based on comparative morphoanatomy. For example, the conifer *Pseudotsuga* displays a gradient in sclereid production on each annual growth increment that has been attributed to shifting auxin levels; copious branching sclereids occur in the basal leaves but decline to few or none in the leaves closest to the stem apex (Al-Talib and Torrey, 1961).

Based on a combination of morphology and anatomy, some leaf fossils are readily assignable to the family Araucariaceae, sometimes even to a particular extant genus (Hill *et al.*, 2008; Pole, 2008). The extinct Late Cretaceous fossil conifer *Brachyphyllum* possessed cyclocytic stomata resembling those of some Araucariaceae, with various modes of orientation in the same leaf, from mostly transverse (within the same cell file) to oblique or almost longitudinal in other cell files (Kunzmann *et al.*, 2004; Kvaček, 2007). Other fossil conifers tentatively assigned to Araucariaceae, such as *Allocladus*, *Geinitzia* and *Pagiophyllum*, also had cyclocytic stomata that were often oriented transversely or obliquely (Raubeson and Gensel, 1991; Jansson *et al.*, 2008; Li *et al.*, 2014). Conversely, some other fossil conifer leaves with transverse or oblique cyclocytic stomata have been tentatively compared with modern Cupressaceae; they include the Tertiary fossil *Cunninghamiostrobus* and the Jurassic fossil *Sewardiodendron* (Miller, 1990; Yao *et al.*, 1998).

#### Krassilovia *clade and other Cretaceous seed plants.*

Herrera *et al.* (2020) identified a clade of extinct Early Cretaceous seed plants that they informally designated the *Krassilovia* clade, including the genera *Cycadocarpidium*, *Krassilovia*, *Podozamites* and *Swedenborgia*, although these names represent organ genera, potentially of the same taxon. The group is partly characterized by stomata that consistently exhibit a transverse orientation (Shi *et al.*, 2018; Herrera *et al.*, 2020). These plants differed from modern Araucariaceae in several respects. The leaves were mostly linear, broad and strap-shaped, with several parallel veins, compared with often short, triangular and imbricate leaves in Araucariaceae. The stomata lacked the distinctive ring of subsidiary cells that characterize all Araucariaceae, and instead either lacked subsidiary cells entirely or possessed a pair of LSCs in the same cell file as the GCs, rather similar to the arrangement illustrated here in some angiosperms, such as *Cassytha* and *Casuarina* (Fig. 3A, B, F).

A broadly similar stomatal structure occurs in *Cearania*, another Early Cretaceous seed plant, which has been assigned tentatively to Gnetales, partly due to its herbaceous or shrubby habit (Kunzmann *et al*., 2009, 2011). *Cearania* differed morphologically from the *Krassilovia* clade; the leaves were variable in shape but relatively small and thick, although with parallel venation. They possessed transverse (or oblique) stomata arranged in longitudinal files of axially elongated epidermal cells, either lacking subsidiary cells or with a pair of LSCs in the same cell file as the GCs. Kunzmann *et al.* (2009) compared *Cearania* with the taxonomically isolated extant genus *Ephedra* (Gnetales). They noted that although many species of *Ephedra* have longitudinal stomatal orientation, in *Ephedra trifurca* the stomata show unusually variable GC orientation: transverse or oblique or, more rarely, longitudinal. Likewise, in *Novaolindia*, an Early Cretaceous gymnosperm of unknown affinity, the stomata were mostly transverse relative to the axis (Kunzmann *et al.*, 2007).

#### Bennettitales.

The seed-plant order Bennettitales (syn. Cycadeoidales) was relatively diverse and abundant during the Mesosoic era, until it became extinct in the Cretaceous period. A highly diagnostic feature of the order is the presence of transverse stomata with a distinct pair of LSCs (Fig. 4A–D), typically associated with distinctive GC thickenings and undulating anticlinal walls on intercostal epidermal cells (Florin, 1933; Sincock and Watson, 1988; Watson and Sincock, 1992; Rudall *et al.*, 2013; Rudall and Bateman, 2019). For example, *Zamites* (Bennettitales) had compound leaves with strap-shaped pinnae bearing transverse stomata in axial cell files (Kvaček, 2022). Stomatal orientation can vary from random to transverse across a single pinna in bennettite leaves (Rudall and Bateman, 2019). This variation could be related to the venation pattern, which is relatively diverse in bennettites, ranging from predominantly parallel to reticulate (Watson and Sincock, 1992).

## CONCLUSIONS

In most land plants, the GCs control gas exchange by adjustments in shape achieved by differences in turgor pressure that lead to opening and closure of the pore. At least in seed plants, such movements are associated with metabolic exchange of potassium ion concentrations with surrounding cells, given that augmented levels of abscisic acid can trigger osmotic ion efflux (Bauer *et al.*, 2013; Brodribb *et al.*, 2020). However, studies of stomatal physiology and ionic balance have focused primarily on angiosperms; preliminary comparative investigation of a conifer species (*Metasequoia glyptostroboides*) have tentatively suggested a less active role for abscisic acid in gymnosperms (McAdam and Brodribb, 2014).

In many angiosperms, biomechanical movements of GCs are facilitated by the unique radial orientation of the wall microfibrils, which restrict radial movement and thus cause longitudinal expansion and bending of the pore (Payne, 1979; Huang and Wang, 1997; Shtein *et al.*, 2017; Woolfenden *et al.*, 2018). Grass GCs appear to be unique in possessing longitudinal organization of the cellulose microfibrils (Spiegelhalder and Raissig, 2021). Longitudinal orientation of the GCs maximizes surface contact with neighbouring cells in adjacent cell files to facilitate passage of osmotic substances. This effect is intensified in grasses, in which the LSCs are highly modified and have a demonstrable physiological role in ion exchange and stomatal movements (Franks and Farquhar, 2007).

Despite these apparent benefits of longitudinal GCs and the strong developmental constraints that accompany stomatal development, why did transverse stomata apparently evolve iteratively in a few seed-plant groups during the Mesozoic era? And why do some parasitic or xerophytic angiosperms display this otherwise rare character? Parasitic plants have a higher transpiration rate than their host plants, a physiological necessity that facilitates passage of water from the host to the parasite. This aspect has been investigated most intensively in the hemiparasitic mistletoe genus *Viscum* (Viscaceae), which has transverse stomata that remain open. As suggested by Wilson and Calvin (2003), a well-developed cuticular epithelium in *Viscum* could therefore represent an alternative strategy to restrict water evaporation. In *Cassytha*, haustorial access to the host plant occurs even before stomatal differentiation (Heide-Jørgensen, 1991), and its transversely oriented stomata are probably subfunctional. The parasitic habit probably evolved initially to overcome water deficiency in some land plants, and thus, arguably, itself represents a form of xeromorphy. Ecological factors, such as the Cretaceous CO_2_ decline and changing water availability, could have played a part in driving experimentation in leaf shapes and reduced leaves with denser venation (de Boer *et al.*, 2012). Additional factors could include experimentation in stomatal development that sometimes accompanied the transition to a hemiparasitic habit.

The variability of transverse (or oblique) stomata in some gymnosperms, such as living *Wollemia* and extinct *Cearania*, suggests that this feature is linked to highly localized contrasts in auxin levels at critical developmental stages during leaf expansion. However, it appears to present a more stable picture in the few angiosperm groups that possess this feature. Development of the venation pattern and stomatal orientation during leaf expansion are interconnected auxin-driven processes, and their functional coordination has a genetic basis (Sack and Scoffoni, 2013). Given the demonstrable finely-tuned roles of auxin levels at different stages in stomatal development, it is likely that transverse stomata arose in different groups as a non-lethal developmental shift that subsequently became more or less fixed during evolution in some taxa, especially among angiosperms.
